# Rs4612666 Polymorphism of the NLRP3 Gene Is Associated with the Occurrence of Large Artery Atherosclerotic Ischemic Strokes and Microembolic Signals

**DOI:** 10.1155/2018/6345805

**Published:** 2018-04-23

**Authors:** Lufeng Cheng, Ruihua Yin, Shaonan Yang, Xudong Pan, Aijun Ma

**Affiliations:** Department of Neurology, The Affiliated Hospital of Qingdao University, 59 Haier Road, Qingdao, Shandong 266000, China

## Abstract

**Purpose:**

Large artery atherosclerosis (LAA) ischemic stroke (IS) is the most common IS subtype, and microemboli are clinically important for indicating an increased risk of IS. Nucleotide-binding domain-like receptor protein 3 (NLRP3) plays a crucial role in the pathogenesis of atherosclerosis. The aim of this study is to investigate the relationship between NLRP3 gene polymorphisms and susceptibility for LAA IS and microembolic signals (MES) in the Chinese Han population.

**Methods:**

We studied 293 patients diagnosed with LAA IS and 265 controls. Transcranial Doppler (TCD) was used to monitor the MES in all of the patients. Depending on the presence or absence of MES, the patients were divided into MES-positive and MES-negative subgroups. PCR-RFLP or direct sequencing were used to analyze three NLRP3 gene polymorphisms.

**Results:**

Seventy-six patients presented with MES and the MES-positive rate was 25.94%. Logistic regression analysis showed that the TT genotype frequency for the rs4612666 gene polymorphism was higher in study patients than in the controls (adjusted *P* = 0.001) and higher in MES-positive patients compared to MES-negative patients (adjusted *P* = 0.015). The T allele of rs4612666 was associated with an increased risk for developing LAA IS and MES (*P* = 0.001; *P* = 0.015, resp.). Prevalence of the CCC haplotype was higher in the controls than in the patients (*P* = 0.009) and prevalence of the TGT haplotype was lower in the controls than in the patients (*P* = 0.019).

**Conclusions:**

The NLRP3 rs4612666 gene polymorphism may be related to the occurrence of LAA IS and MES, suggesting that the NLRP3 gene polymorphism increases the susceptibility of LAA IS by changing the plaque vulnerability.

## 1. Introduction

A systematic analysis for the Global Burden of Disease Study (GBD) 2015 reported that, among all of the neurological disorders analyzed, strokes account for the largest proportion of total global disability adjusted life-years (DALYS) (47.3%) and deaths (67.3%) [1]. Ischemic stroke (IS) accounts for 65%–80% of new increased stroke cases [2]. IS is believed to be a multifactorial disorder with many elements, including environmental and genetic factors associated with its pathogenesis [3]. According to the TOAST (Trial of ORG 10172 in Acute Stroke Treatment) system, large artery atherosclerosis (LAA) ischemic strokes are the most common stroke subtype [4] and are more likely to be influenced by genetic profiles [5]. Therefore, the study of gene polymorphisms and their relationship with LAA IS is of great importance for the prevention, diagnosis, and treatment of IS.

In recent years, the role that inflammation plays in atherosclerosis and its complications has drawn considerable attention [6]. The current understanding of the importance of inflammation during all phases of atherosclerosis, including formation, progression, and rupture of atherosclerotic plaques, has greatly increased [7]. In patients with large artery occlusive disease, active microemboli are released from the unstable atherosclerotic lesions and lead to acute cerebral infraction. Studies have shown that the presence of microembolic signals (MES) detected by transcranial Doppler ultrasound (TCD) is potential risk markers for IS and is a significant sign of instability in the atherosclerotic plaque [8].

The nucleotide-binding domain-like receptor protein 3 (NLRP3) inflammasome is the most well-understood inflammasome and plays an important role in the process of inflammation. The NLRP3 inflammasome is composed of NLRP3, the adaptor protein, apoptosis-associated speck-like protein (ASC), and proinflammatory caspase-1. Upon activation of NLRP3 by pathogen-associated molecular patterns (PAMPs) and damage-associated molecular patterns (DPMPs), NLRP3 assembles and leads to the excretion of mature proinflammatory cytokines, such as IL-1*β* and IL-18 [9]. Therefore, the NLRP3 inflammasome plays an important role in the inflammatory process of atherosclerosis. The NLRP3 gene was identified as the genetic locus for three dominantly inherited periodic fevers: familial cold autoinflammatory syndrome (FCAS), Muckle-Wells syndrome [10], and chronic infantile neurological cutaneous articular syndrome/neonatal onset multisystem inflammatory disease (CINCA/NOMID) [11]. These three diseases are collectively known as cryopyrin-associated periodic syndrome (CAPS), suggesting that the disease-associated variants of NLRP3 probably encode a hyperactive version of NLRP3 which promotes excessive production of IL-1*β* [12]. NLRP3 gene mutations are also observed to be associated with rheumatoid arthritis [13], Crohn's disease [14], and abdominal aortic aneurysms [15]. One study from China suggested that genetic polymorphisms in NLRP3 may influence the risk of ischemic stroke in the Chinese population [16]. To the best of our knowledge, there are limited studies regarding the relationship between NLRP3 polymorphisms and susceptibility for LAA stroke and MES in the Chinese Han population. Therefore, we performed transcranial Doppler emboli detection and genotyped the NLRP3 gene polymorphisms to investigate whether NLRP3 gene polymorphisms are associated with LAA stroke and MES.

## 2. Materials and Methods

### 2.1. Study Population

All recruited subjects were unrelated ethnic Han Chinese. We studied 293 patients who were diagnosed with LAA strokes according to the TOAST classification system [4] and had lesions that were limited to the internal carotid artery or middle cerebral artery regions. All patients were hospitalized in the Neurology Department of Affiliated Hospital of Qingdao University from January 2014 to December 2016. All patients were subjected to microemboli-monitoring. occurrence of MES, the patients were divided into MES-positive and MES-negative groups. Inclusion criteria for stroke patients were cerebral infarction confirmed by computer tomography (CT) or magnetic resonance (MR), cerebral vascular and cardiac lesions confirmed by TCD, cardiac ultrasound, brain magnetic resonance angiography (MRA), or whole brain digital subtraction angiography (DSA). Exclusion criteria were patients who were diagnosed with any other subtype of IS (cardioembolism, small-vessel occlusion, stroke of other determined etiology, and stroke of undetermined etiology) or had severe heart disease (recent myocardial infarction, angina pectoris disease, and valvular heart disease) and severe infection, severe liver and renal dysfunction, and cancer. Two hundred sixty-five controls were recruited from the Health Examination Center. Inclusion criteria were the following: the subjects had no history of ischemic stroke, no cerebral infarction by brain CT or MRI, and no obvious atherosclerosis or angiostenosis by TCD, CTA, or MRA. Exclusion criteria were the same as the criteria used for the patient group.

The research was performed in accordance with the Code of Ethics of the World Medical Association (Declaration of Helsinki) for experiments involving human subjects. The study was permitted by the ethical committee of the Affiliated Hospital of Qingdao University (QDDXYXYFSXY-2014-005). Informed consent was obtained from all participants.

### 2.2. Clinical Measurements and Laboratory Analysis

Collection of clinical data from the subjects was performed by well-trained investigators. Questionnaire surveys were conducted to investigate the general status of the subjects, including gender, age, history of hypertension, diabetes, coronary artery disease (CAD), history of smoking and drinking, BMI, and family history of cerebrocardiovascular events. Blood samples (4 ml) were collected from the antecubital vein of all participants following an overnight fast and later added to EDTA tubes followed by a 10-minute centrifugation at 3000 rpm. Levels of serum high sensitivity C-reactive protein (hs-CRP), total cholesterol (TC), triglycerides (TG), high-density lipoproteins (HDL), low-density lipoproteins (LDL), and blood glucose (GLU) were measured in the laboratory of our hospital.

### 2.3. Genotyping the Polymorphisms

**Figure 1:**
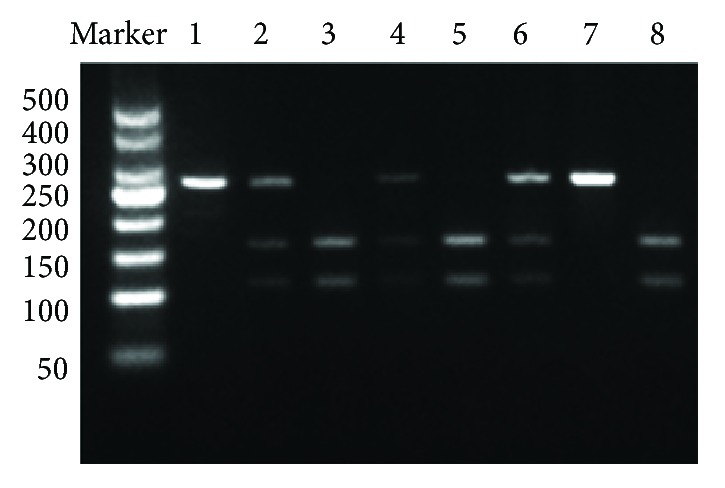
Agarose gel electrophoresis of the rs4612666 polymorphism. Line 1: TT; line 2: CT; line 3: CC; line 4: CT; line 5: CC; line 6: CT; line 7: TT; line 8: CC.

**Figure 2:**
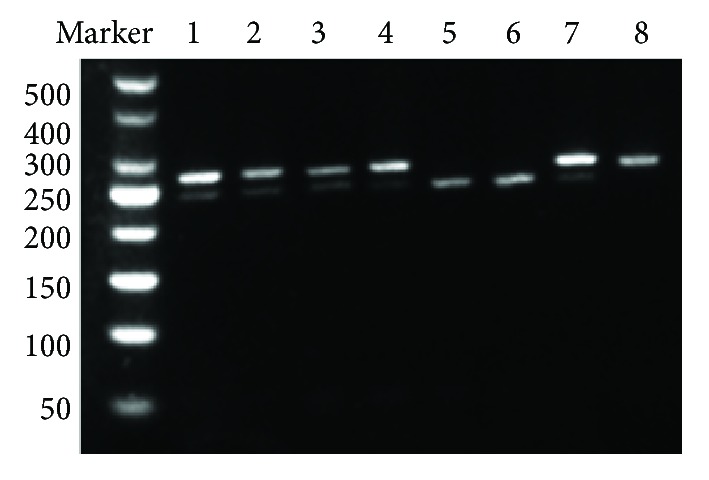
Agarose gel electrophoresis of the rs10754558 polymorphism. Line 1: CG; line 2: CG; line 3: CG; line 4: CG; line 5: CC; line 6: CC; line 7: CG; line 8: GG.

**Figure 3:**
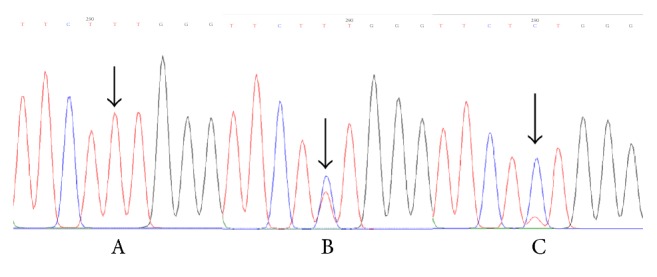
Sequencing of rs7512998. A: TT; B: CT; C: CC.

Genomic DNA was extracted from peripheral blood leukocytes using the TIANamp Blood DNA kit (Tiangen Biotech, Beijing, China) according to the manufacturer's instructions. Polymerase chain reaction and restriction fragment length polymorphism (PCR-RFLP) testing was used to investigate the NLRP3 rs4612666 and rs10754558 gene polymorphisms. Primers were designed and synthesized by Ruibiotech (Beijing, China). PCR amplifications were performed in a total reaction volume of 20 *μ*l that contained 2 *μ*l genomic DNA, 10 *μ*l 2XTaq MasterMix, 0.5 *μ*l of both forward and reverse primers (10 *μ*mol/L), and 7 *μ*l double-distilled H2O. The PCR amplification conditions were as follows: initial denaturation at 95°C for 5 min, followed by 30 cycles at 95°C for 30 s, annealing at 57°C (or 58°C) for 30 s, and extension at 72°C for 30 s followed by a final extension step at 72°C for 7 min. After PCR amplification, 3.5 *μ*l of PCR product was digested by restriction enzyme (New England Biolabs, Beijing, China) for 0.5 h at 37°C and subsequently evaluated by gel electrophoresis on a 2% agarose gel. Photographs (Figures 1 and 2) were taken using an automatic fluorescence/chemiluminescence imaging analysis system (Villber Luomat FX-X20 M). The rs7512998 genotype (Figure 3) was analyzed by direct sequencing (Ruibiotech, Beijing, China). The primer sequences and restriction enzymes are shown in Table 1.

### 2.4. Microembolic Signal Monitoring

TCD (EMS-9EB × 2P Doppler box/EMS-9EB multidrop × 4.2 MHz probe) was used to monitor the MES in all patients within 72 h of stroke onset. The 2 MHz probe was fixed to the patients' head, and the MES monitoring was performed in the initial segment and distal segment of the symptomatic middle cerebral artery (MCA) (sampling depth, 50–65 mm; distance between two points, ≥6 cm; sample volume, 8–15 cm; MES relative threshold, ≥5 dB; and duration of microemboli-monitoring, 60 min). Microemboli monitoring was performed by a system-trained professional and identified by two experienced neurology physicians. The criteria for microembolic signals [17] were (1) transient microembolic signal (<300 ms); (2) high-intensity signal (≥7 dB above the background signal); (3) being unidirectional within the Doppler velocity spectrum; (4) signals emerging randomly during the cardiac cycle; and (5) an audible sound (click, chirp, and whistle).

### 2.5. Statistical Analysis

Statistical analyses were performed using SPSS 20.0. We calculated allele frequencies and tested for agreement with the Hardy-Weinberg equilibrium using a Chi-square test for each locus. Continuous variables were shown as the mean ± standard deviation (SD), and Student's *t*-test was used to analyze the differences. Categorical variables were presented as a frequency, and a Chi-square test was performed. Comparisons of genotype and allele frequencies were analyzed by Pearson's *χ*^2^ test or Fisher's exact test. Logistic regression analyses were used to calculate odds ratios (OR) with 95% confidential intervals (CI) after an adjustment with covariates. The SHEsis software platform was used for analyses of linkage disequilibrium (LD) and haplotype distributions [18, 19]. A *P* value of less than 0.05 was considered to be statistically significant.

## 3. Results

### 3.1. Clinical Characteristics of LAA and Control Groups

Table 2 shows the clinical characteristics of the LAA stroke patients and control subjects. There were no significant differences between the two cohorts in terms of age, history of CAD, BMI, and the levels of LDL (*P* > 0.05). The frequency of males, patients with hypertension and diabetes, smoking, alcohol drinking, family history of cerebrocardiovascular events, and the level of TGs, TC, LDL, hs-CRP, and GLU were higher in stroke groups than in control groups (*P* < 0.05). The HDL levels were lower in stroke groups compared to control groups (*P* < 0.05).

### 3.2. Association between NLRP3 Gene Polymorphisms and LAA Strokes

Genotype distributions did not deviate from the Hardy-Weinberg equilibrium in both groups (*P* > 0.05). When compared to the reference (CC), we observed that the TT genotype of rs4612666 was associated with a significantly increased risk of LAA stroke after controlling for other covariates selected in our study (adjusted OR = 3.021, 95% CI 1.593–5.731, *P* = 0.001). Furthermore, patients carrying the T allele showed an increased risk of stroke compared with carriers of the C allele (OR = 1.506, 95% CI 1.188–1.909, *P* = 0.001). However, the genotypes and allele frequencies for rs10754558 and rs7512998 were not significantly different between the two groups (Table 3).

### 3.3. Association between NLRP3 Gene Polymorphisms and Microembolic Signals

Seventy-six patients presented with microembolic signals in the LAA stroke group. There were no significant differences in the clinical characteristics between the MES-positive and MES-negative groups (Table 4). Logistic regression analysis showed that patients carrying the rs4612666 TT genotypes were at a significantly increased risk of microembolic signals (adjusted OR = 2.706, 95% CI 1.210–6.048, *P* = 0.015). Patients carrying the T allele were more likely to have microembolic signals (OR = 1.593, 95% CI 1.095–2.315, *P* = 0.015). There was no association between microembolic signals and the rs10754558 or 7512998 genotypes (Table 5).

### 3.4. Haplotype Analysis

**Figure 4:**
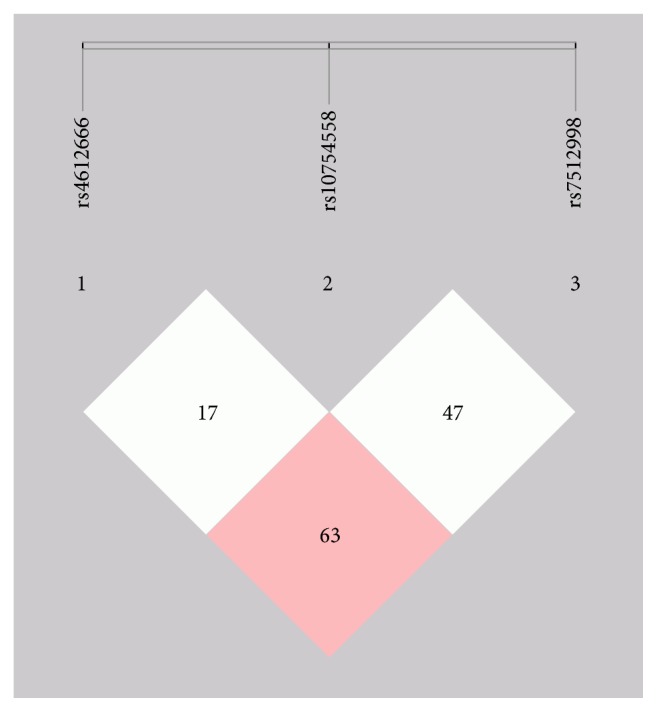
Linkage disequilibrium tests of rs4612666-rs10754558-rs7512998: the *D*′ value between rs4612666 and rs7512998 is 0.63, the *D*^″^ value between rs10754558 and rs7512998 is 0.47, and the *D*′ value between rs4612666 and rs7512998 is 0.17.

Using linkage disequilibrium (LD) test results, we conducted haplotype analyses for three SNPs using the SHEsis software platform (Figure 4). The results showed that frequency of the CCC haplotype in the control group was higher than in the LAA group and frequency of the TGT haplotype in the control group was lower than in the LAA group, indicating that the CCC haplotype may play a protective role against LAA stroke and the TGT haplotype may have the opposite effect. (Table 6).

## 4. Discussion

Our study examining the relationship between SNPs of NLRP3 (rs4612666, rs10754558, and rs7512998) and LAA IS demonstrated that the frequency of the TT genotype and T allele of the NLRP3 rs4612666 polymorphism was considerably higher in the LAA group than in the control group, suggesting that the T allele may be associated with a susceptibility for LAA IS. However, the current study failed to demonstrate any influence that rs10754558 and rs7512998 may have on LAA IS.

NLRP3 is encoded by the NLRP3 gene, which is located on chromosome 1q44. There are more than 1000 SNPs in the human NLRP3 gene. Previous studies have shown that there is an association between NLRP3 gene polymorphisms and susceptibilities to certain diseases, including Crohn's disease [14], abdominal aortic aneurysms [15], and late-onset Alzheimer's disease [20]. The influence of an NLRP3 gene polymorphism on ischemic strokes was also shown in Zhu et al.'s study [16]. LAA ischemic stroke is the most common type of ischemic stroke and is primarily caused by atherosclerosis. Over the past decade, we have demonstrated that inflammation plays a prominent role in atherosclerosis. The effect of the NLRP3 inflammasome cannot be ignored in the process of inflammation. Cholesterol crystals deposited in atherosclerotic lesions are likely essential intermediate steps in activating NLRP3 inflammation and leading to injury of the vascular wall. This mechanism involves both potassium efflux and translocation of lysosomal proteolytic contents [21, 22]. Duewell et al. [21] illustrated that mice with bone marrow-derived cells lacking NLRP3 inflammasome components were markedly resistant to the development of atherosclerosis. Recent studies have revealed involvement of the NLRP3 inflammasome in various human diseases, such as multiple sclerosis (MS) [23], gout [24], type 2 diabetes [25], and inflammatory bowel diseases [26]. This finding suggests that NLRP3 gene mutations may be related to the occurrence of different inflammatory diseases. Hitomi et al. [27] reported that there are significant associations between the human NLRP3 polymorphism rs4612666 and susceptibility to food-induced anaphylaxis and aspirin-induced asthma (AIA). The mechanism by which this occurs may be that the variant influences higher mRNA expression by altering expression enhancer activity or mRNA stability. One study by Zheng et al. [28] showed that there was no relationship between the rs4612666 polymorphism and type 2 diabetes and insulin resistance. In a novel finding, this study has shown a significant relationship between the rs4612666 polymorphism and LAA ischemic stroke. Our study revealed that the TT genotype increases the risk of LAA ischemic stroke (OR = 3.021), and the T allele is also an independent risk factor (OR = 1.506). This suggests that the rs4612666 TT genotype and T allele are associated with a susceptibility for LAA strokes in the Chinese Han population. However, we did not perform any detailed experiments to investigate serum levels of the NLRP3 inflammasome, mRNA, IL-18, and IL-1*β*. Therefore, the influence that rs4612666 has on NLRP3 inflammasome expression remains somewhat a mystery. Further studies investigating the mechanisms of NLRP3 gene polymorphisms on atherosclerosis are necessary.

Numerous studies have demonstrated the association between the NLRP3 rs10754558 polymorphism and a susceptibility for inflammatory diseases. Zhou et al. [16] reported that the G allele for NLRP3 rs10754558 was associated with an increased risk for ischemic stroke. Similarly, the G allele has been reported to be correlated with CAD [29], type 2 diabetes [28], and food-induced anaphylaxis and aspirin-induced asthma [27]. However, one particular meta-analysis has suggested there is an association between the NLRP3 rs10754558 C allele and autoimmune and inflammatory diseases in the Latin American population but not the European and Asian ones [30]. The C allele seems to be protective against HIV-1 infection [31]. The present study demonstrates that the NLRP3 rs10754558 gene polymorphism has no relationship to LAA strokes, differing from the results of Zhu et al. This finding may be observed because the polymorphism's allelic distribution varies with the ethnic origin of the studied population. There was also no ischemic stroke subtype division in Zhu et al.'s study. There was limited investigation into NLRP3 rs7512998. Kunnas et al. [32] reported that the NLRP3 gene polymorphism rs7512998 C allele was associated with higher systolic and diastolic blood pressure in 50-year-old subjects. However, no relationship was found between rs7512998 and type 2 diabetes mellitus in a study by Wang et al. [33]. No significant association was found between rs7512998 and LAA strokes in our study. Additional linkage disequilibrium analysis and haplotype analysis suggested that the three SNPs were in incomplete linkage disequilibrium. The frequency of the CCC haplotype in the LAA group was significantly lower than in the control group and frequency of the TGT haplotype in the LAA group was higher than in the control group, suggesting that the CCC haplotype may play a protective role against LAA stroke and the TGT haplotype may have the opposite effect.

To investigate whether the NLRP3 gene polymorphism stabilizes atherosclerotic plaques, we used TCD to monitor MES in our patients. In our study, 76 patients presented with MES, which was a prevalence of 25.94%. This was a lower prevalence than what was reported by Jiang and Iguchi et al. In a study by Jiang et al., 15 patients presented with MES out of 49 LAA patients who underwent microemboli-monitoring within 48 h of stroke onset. The MES rate for this study was 30.61% [34]. Iguchi et al. reported an MES-positive rate of 49% within 24 h of stroke onset [35]. The reason for our lower rate of MES may be because our microemboli-monitoring was performed within 72 h of stroke onset, and antiplatelet therapy was given to every patient before monitoring. It has been revealed that MES-positive rates can be reduced with antithrombotic therapy and longer periods of time between symptom onset and monitoring [36, 37]. Further analysis showed that patients carrying the T allele were more likely to have MES, demonstrating that the NLRP3 rs4612666 polymorphism was involved in the MES occurrence. This occurrence of microemboli may be due to two mechanisms: the rupture of atherosclerotic plaque contents into the blood stream, and the breaking off of a thrombus formed on an ulcerated surface or in the bloodstream [38]. MES indicates that the plaque is unstable. The break off of plaque is highly associated with inflammation. Varghese et al. reported that expression of NLRP3 mRNA was markedly higher in plaques of symptomatic patients than in asymptomatic patients, indicating that the activation of the NLRP3 inflammasome is related to plaque vulnerability. The NLRP3 gene variants changed the mRNA and expression levels of related inflammatory mediators [39]. Therefore, we hypothesize that the rs4612666 polymorphism leads to the destabilization of atherosclerotic plaque by accelerating inflammation in a lesion.

Our study indicates that the NLRP3 gene polymorphism rs4612666 may be associated with the occurrence of LAA ischemic strokes and MES in the Chinese Han population, suggesting that the NLRP3 gene polymorphism influences the susceptibility of LAA IS by changing plaque vulnerability. However, the present study contains a relatively low number of individuals, which may be a limitation. Additionally, there were only three gene polymorphism loci analyzed, which does not represent the entire gene. The replication of this research in different populations and additional in vivo analysis are required to completely elucidate the roles by which NLRP3 polymorphisms predispose for LAA strokes and MES.

## Figures and Tables

**Table 1:** Primer sequence and restriction enzyme.

SNP	Primer sequence (5′ → 3′)	Annealing temperature (°C)	Restriction enzyme	Fragment length (bp)
rs4612666	F: TGCTTAAGGCCATTAATTGTG R: CTCCACCATGGACAAGGAAG	57	BbsI	TT: 260 TC: 260, 102, 105 CC: 102, 105
rs10754558	F: CCGGGCATGGTGGCTCA R: CCCCCGGCTGCACACTG	58	MboI	GG: 261 GC: 261, 236, 25 CC: 236, 25
rs7512998	F: GTAACCACCATTCTATTTGC R: CTGTTGGTGGGAAATGGTGC	58	Direct sequencing	

**Table 2:** Clinical characteristics of LAA patients and control subjects.

Variables	LAA group (*n* = 293)	Control group (*n* = 265)	*P* value
Age (years)	62.89 ± 12.58	61.27 ± 10.32	0.097
Sex (man,%)	219 (74.7%)	150 (56.6%)	<0.001
Hypertension (*n*, %)	200 (68.3%)	122 (46.0%)	<0.001
Diabetes (*n*, %)	86 (29.4%)	46 (17.4%)	0.001
CAD (*n*, %)	68 (23.2%)	65 (24.5%)	0.715
Smoking (*n*, %)	143 (48.8%)	54 (20.4%)	<0.001
Drinking (*n*, %)	121 (41.3%)	56 (21.1%)	<0.001
Family history of cerebro-cardiovascular events (*n*, %)	61 (20.8%)	19 (7.2%)	<0.001
BMI (kg/m^2^)	23.98 ± 3.96	23.87 ± 3.73	0.727
TG (mmol/L)	1.56 ± 0.89	1.79 ± 1.09	0.008
TC (mmol/L)	4.46 ± 1.14	4.80 ± 0.98	<0.001
HDL (mmol/L)	1.05 ± 2.33	1.24 ± 0.49	<0.001
LDL (mmol/L)	2.70 ± 0.87	2.60 ± 0.69	0.135
hs-CRP (mmol/L)	7.58 ± 9.46	2.33 ± 2.04	<0.001
GLU (mmol/L)	6.27 ± 2.51	5.46 ± 1.50	<0.001

CAD: coronary artery disease; BMI: body mass index; TG: triglycerides; TC: total cholesterol; HDL: high-density lipoprotein; LDL: low-density lipoprotein; Hs-CRP: high-sensitivity C-reactive protein; GLU: fasting blood-glucose.

**Table 3:** Genotype and allelic frequencies of NLRP3 SNPs in LAA patients and control subjects.

SNP site	LAA group (%)	Control group (%)	*P* value	Adjusted OR	95% CI
rs4612666					
genotype					
CC	63 (21.5%)	93 (35.1%)	-	1	
CT	163 (55.6%)	129 (48.7%)	0.090	1.531	0.935–2.505
TT	67 (22.9%)	43 (16.2%)	0.001	3.021	1.593–5.731
allele					
C	289 (49.3%)	315 (59.4%)	-	1	
T	297 (50.7%)	215 (40.6%)	0.001	1.506	1.188–1.909
rs10754558					
genotype					
CC	73 (24.9%)	71 (26.8%)	-	1	
CG	154 (52.6%)	131 (49.4%)	0.650	1.127	0.672–1.889
GG	66 (22.5%)	63 (23.8%)	0.538	1.184	0.648–2.163
Allele					
C	300 (51.2%)	273 (51.5%)	-	1	
G	286 (48.8)	258 (48.5%)	0.916	1.013	0.801–1.281
rs7512998					
genotype					
CC	2 (0.7%)	3 (1.1%)	-	1	
CT	45 (15.4%)	56 (21.1%)	0.492	2.275	0.218–23.768
TT	246 (84.0%)	206 (77.7%)	0.278	3.584	0.358–35.896
allele					
C	49 (8.4%)	62 (11.7%)	-	1	
T	537 (91.6%)	468 (88.3%)	0.063	1.452	0.978–2.154

**Table 4:** Clinical characteristics of MES(+) and MES(−) groups.

variables	MES(+) (*n* = 76)	MES(−) (*n* = 217)	*P* value
Age (years)	63.79 ± 12.81	62.58 ± 12.51	0.470
Sex (man, %)	55 (72.4%)	164 (75.6%)	0.580
Hypertension (*n*, %)	46 (60.5%)	154 (71.0%)	0.092
Diabetes (*n*, %)	21 (27.6%)	65 (30.0%)	0.702
CAD (*n*, %)	18 (23.7%)	50 (23.0%)	0.909
Smoking (*n*, %)	36 (47.4%)	107 (49.3%)	0.771
Drinking (*n*, %)	27 (35.5%)	94 (43.3%)	0.235
Family history of cerebrocardiovascular events (*n*, %)	16 (21.1%)	45 (20.7%)	0.954
BMI (kg/m^2^)	24.32 ± 3.95	23.87 ± 3.96	0.387
TG (mmol/l)	1.45 ± 0.56	1.60 ± 0.98	0.102
TC (mmol/l)	4.53 ± 0.98	4.44 ± 1.19	0.551
HDL (mmol/L)	1.04 ± 0.25	1.05 ± 0.23	0.644
LDL (mmol/L)	2.75 ± 0.74	2.68 ± 0.91	0.560
hs-CRP (mmol/L)	7.51 ± 10.22	7.60 ± 9.20	0.946
GLU (mmol/L)	6.19 ± 2.70	6.30 ± 2.45	0.746

**Table 5:** Genotypes and allelic frequencies of NLRP3 SNPs in MES(+) and MES(−) groups.

SNP site	MES(+) (%)	MES(−) (%)	*P* value	OR	95% CI
rs4612666					
genotype					
CC	12 (15.8%)	51 (23.5%)	-	1	
CT	38 (50.0%)	125 (57.6%)	0.464	1.314	0.633–2.730
TT	26 (34.2%)	41 (18.9%)	0.015	2.706	1.210–6.048
allele					
C	62 (40.8%)	227 (52.3%)	-	1	
T	90 (59.2%)	207 (47.7%)	0.015	1.593	1.095–2.315
rs10754558					
genotype					
CC	18 (23.7%)	55 (25.3%)	-	1	
CG	42 (55.3%)	112 (51.6%)	0.624	1.175	0.617–2.237
GG	16 (21.1%)	50 (23.0%)	0.841	1.084	0.494–2.378
allele					
C	78 (51.3%)	222 (51.2%)	-	1	
G	74 (48.7%)	212 (48.8%)	0.972	0.993	0.687–1.438
rs7512998					
genotype					
CC	1 (1.3%)	1 (0.5%)	-	1	
CT	15 (19.75%)	30 (13.8%)	0.521	0.388	0.022–6.999
TT	60 (78.95%)	186 (85.7%)	0.318	0.234	0.014–4.047
allele					
C	17 (11.2%)	32 (0.074)	-	1	
T	135 (88.8%)	402 (0.926)	0.144	0.632	0.340–1.175

**Table 6:** Haplotype analysis of rs4612666-rs10754558-rs7512998 in LAA patients and control subjects.

haplotype	LAA group (freq)	Control group (freq)	*P* value	OR	95% CI
C C C	26.52 (0.045)	44.38 (0.084)	0.009	0.521	0.317–0.856
C C T	98.42 (0.168)	95.45 (0.180)	0.620	0.924	0.677–1.262
C G C	7.81 (0.013)	15.02 (0.028)	-	-	-
C G T	156.24 (0.267)	160.15 (0.302)	0.205	0.844	0.649–1.097
T C C	11.15 (0.019)	0.08 (0.000)	-	-	-
T C T	163.91 (0.280)	133.09 (0.251)	0.254	1.169	0.894–1.529
T G C	3.52 (0.006)	2.51 (0.005)	-	-	-
T G T	118.42 (0.202)	79.31 (0.150)	0.019	1.453	1.062–1.988

All those frequencies < 0.03 were ignored in analysis.
